# Editorial: Recent Advances in Bioremediation/Biodegradation by Extreme Microorganisms

**DOI:** 10.3389/fmicb.2019.01851

**Published:** 2019-08-14

**Authors:** Edgardo Rubén Donati, Rajesh Kumar Sani, Kian Mau Goh, Kok-Gan Chan

**Affiliations:** ^1^Facultad de Ciencias Exactas, CINDEFI (CCT, La Plata-CONICET, UNLP), Universidad Nacional de la Plata, La Plata, Argentina; ^2^Department of Chemical and Biological Engineering, South Dakota School of Mines and Technology, Rapid City, SD, United States; ^3^Faculty of Sciences, Universiti Teknologi Malaysia, Skudai, Malaysia; ^4^Division of Genetics and Molecular Biology, Faculty of Science, Institute of Biological Sciences, University of Malaya, Kuala Lumpur, Malaysia; ^5^International Genome Centre, Jiangsu University, Zhenjiang, China

**Keywords:** pollutants, bioremediation, biodegradation, biomineralization, extremophiles

To remediate polluted sites, biological processes have many advantages from economic, environmental, and practical aspects. Adsorption and biodegradation of organic contaminants and the immobilization, mobilization, and/or transformation of metal(loid)s are the main remediation processes that can be mediated by the action of several microorganisms especially those extremophiles surviving in hostile environments with high concentrations of pollutants. The aim of this Research Topic of Frontiers in Microbiology is to provide an appropriate platform to publish the latest results on the bioremediation of various pollutants by extremophilic pure cultures or microbial consortia. This Research Topic consists of 4 reviews and 7 original articles.

Marques reviewed the theme of extremophiles as microfactories which are able to provide genetic or metabolic mechanisms as controlled services to the clean-up of environmental pollution. The review focuses on metal and radionuclides pollution, and includes a discussion about the use of synthetic biology to improve the bioremediation processes.

Two articles in this Research Topic are focused on heavy metal(loid)s contaminants. Figueroa et al. described that several microorganisms exhibited high resistance to 19 metal(loid)s. Most of those strains displayed metal or metalloid reducing activity, and have been successfully used for the biological synthesis of nanostructures containing metal(loid)s. Tellurium and gold nanostructures showed antibacterial properties, which inhibited *E. coli* and *L. monocytogenes* growth. Acid mine drainage (AMD) is considered a severe environmental problem provoked by the microbial oxidation of sulfidic minerals. Gupta et al. explored the abundance and role of indigenous microorganisms displaying sulfate- and metal(loid)- reducing activity in the natural attenuation of an AMD impacted soil (AIS). The addition of nutrients (e.g., cysteine and lactate) to AIS increased the activity of such microorganisms achieving an increase in pH from 3.5 to 6.6, and reduction of sulfate (95%), iron (50%), and other heavy metals. In this way, Gupta et al. demonstrated that addition of nutrients could biostimulated the growth of some members of phylum Firmicutes (e.g., sulfate- and iron- reducing microorganisms) and bioremediate AMD impacted sites.

Orellana et al. reviewed extensively the most recent research on polyextremophilic microorganisms isolated from a wide range of extreme environments including salars, geothermal springs, deserts, ice fields, and diverse zones in Chile such as Altiplano, Atacama Desert, Central Chile, Patagonia, and Antarctica. This review also discussed the molecular and physiological capabilities of many of these isolates which were beneficial for bioremediation processes.

Diverse anthropogenic activities, particularly the emission due to the burning of fossil fuels, have triggered an alarming rise of CO_2_ in the environment. A description of the measures of greenhouse gases emission is reviewed by Bose and Satyanarayana. In this review, authors discussed the merits and demerits of various approaches with extensive bibliographical material. Finally, a deep description of the use of carbonic anhydrases (CA) for biomineralization of CO_2_ was included. This methodology was proposed as one of the most economical methods to mitigate global warming.

The other six articles in this Research Topic are related to the bioremediation of organic pollutants. Park and Park described the strategies for alkane degradation under extreme conditions (e.g., low and high temperatures, high salt, and acidic and anaerobic conditions). Alkane degraders seem to possess exclusive metabolic pathways and survival strategies. The thermophilic sulfate-reducing archaeon *Archaeoglobus fulgidus* uses a novel alkylsuccinate synthase for long-chain alkane degradation, and the thermophilic *Candidatus Syntrophoarchaeum butanivorans* anaerobically oxidizes butane via alkyl-coenzyme M formation. In addition to alkane degradation, extremophiles produce energy via the glyoxylate shunt and the Pta-AckA pathway when grown on a diverse range of alkanes under stress conditions.

Blázquez et al. focused on the bioremediation of aromatic compounds such as toluene and xylenes. The degradation of such pollutants is relevant due to their carcinogenic and neurotoxic effects to humans. This article provided evidences that the *bss* and *bbs* genes are not only essential for anaerobic degradation of toluene but also for m-xylene oxidation in the beta-proteobacterium *Azoarcus* sp. The peripheral pathway for the anaerobic oxidation of toluene would consist of an initial activation catalyzed by a benzylsuccinate synthase and a subsequent modified oxidation of benzylsuccinate to benzoyl-CoA and succinyl-CoA (both pathways encoded by *bbs* genes). Su et al. determined the crystal structure of the dibenzothiophene (DBT) sulfone monooxygenase (*BdsA*) from *Bacillus subtilis* at the resolution of 2.2 Å. This is one of the key enzymes in the 4S desulfurization pathway catalyzing the oxidation of DBT sulfone to 2′-hydroxybiphenyl 2-sulfonic acid. The structure of the BdsA-FMN complex at 2.4 Å was also determined. Finally, Su et al. showed that mutations in the residues involved in catalysis or flavin substrate-binding, resulted in a significant loss of enzymatic activity.

Meier et al. studies the catabolism of hydroxylated aromatic acids in *A. adeninivorans* and showed that the genes encoding enzymes involved catabolic pathway of gallic acid are induced using aromatic acid substrates as inducers. Through the construction of gallic acid decarboxylase disruption mutants, the authors showed that gallic acid decarboxylase Agdc1p was the only enzyme responsible for the transformation of gallic acid. They suggest that this enzyme might have useful industrial applications not only in bioremediation processes but also in synthesis of chemicals.

Chandra et al. detected that effluents discharged from the pulp and paper industry contain various refractory and androgenic compounds, even after secondary treatment by activated processes. Most of these compounds are classified as endocrine-disrupting chemicals and are environmental toxicants. This study also assessed the degradability of such compounds by biostimulation. The results suggested that pulp and paper mill wastewater, after this secondary detoxification process, could be safe for disposal.

Lastly, Kirtzel et al. investigated the ability of *Schizophyllum commune* to degrade black slates (e.g., metamorphic rocks rich in sulfides, heavy metals and organic matters). *S. commune* is a filamentous basidiomycete possesses a broad range of enzymes including multicopper oxidases such as laccases and laccase-like oxidases. Both life forms (haploid monokaryotic and mated dikaryotic strains) were able to degrade the slate releasing metals at the same time.

This Research Topic of Frontiers in Microbiology shows how bioremediation by means of extremophiles is an active research theme.

## Author Contributions

All authors listed have made a substantial, direct and intellectual contribution to the work, and approved it for publication.

### Conflict of Interest Statement

The authors declare that the research was conducted in the absence of any commercial or financial relationships that could be construed as a potential conflict of interest.

